# Complications after radiotherapy in patients with Graves’ orbitopathy: A nationwide cohort study

**DOI:** 10.1038/s41433-026-04284-9

**Published:** 2026-02-21

**Authors:** Jooyoung Lee, Hwa Young Ahn, Jung Sun Heo, Mina Kim, Jeong Kyu Lee

**Affiliations:** 1 Department of Applied Statistics, Chung-Ang University, Seoul, Korea; 2 Department of Internal Medicine, Chung-Ang University College of Medicine, Seoul, Korea; 3 Department of Ophthalmology, Chung-Ang University College of Medicine, Seoul, Korea

**Keywords:** Risk factors, Thyroid diseases

## Abstract

**Objective:**

To analyse the incidence of complications following radiotherapy (RT) in patients with Graves’ orbitopathy (GO), particularly focusing on the incidence of radiation retinopathy and associated risk factors.

**Methods:**

Data on diagnosed GO patients who received RT between 2008 and 2018 were obtained from the National Health Insurance Service database. The diagnosis of GO and the administration of RT were assessed utilising the International Classification of Diseases codes. The incidence of RT-related complications was analysed. Logistic regression was used to identify risk factors for radiation retinopathy.

**Results:**

Among the 1108 patients who received RT for GO between 2008 and 2018, 67 patients (6.0%) were newly diagnosed with cataracts, and 42 patients (3.8%) underwent surgery. Ocular surface disease, dry eye syndrome, eyelid inflammation, and head and neck cancer occurred in 14 patients (1.3%), 37 patients (3.3%), 8 patients (0.7%), and 2 patients (0.2%), respectively, and no cases of leukaemia or lymphoma were observed. Radiation retinopathy developed in 63 patients (5.7%). In comparison to those without radiation retinopathy, patients with radiation retinopathy had a short interval from GO diagnosis to RT (odds ratio (OR) = 0.95, 95% CI = 0.92–0.98, *p* = 0.003) and high fasting blood sugar levels (OR = 1.01, 95% CI = 1.00–1.03, *p* = 0.014).

**Conclusions:**

In patients with GO, cataract, radiation retinopathy, dry eye syndrome, ocular surface disease, and head and neck cancers are potential complications of orbital RT. Risk factor identification and long-term monitoring are crucial for minimising treatment-related complications and improving patient outcomes.

## Introduction

Graves’ orbitopathy (GO) is an autoimmune disorder mainly associated with Graves’ disease [1]. It involves inflammatory changes in orbital tissues with the potential thickening and fibrosis of the extraocular muscles and orbital fat, leading to an increase in volume within the bony orbit [2]. It can cause discomfort, visual disturbances, and a significant decrease in quality of life. Treatment approaches vary based on the activity and severity of the disease [3].

Orbital radiotherapy (RT) has been used in the treatment of GO since 1936 and has remained a fundamental therapeutic option for decades [4]. It is generally considered a second-line treatment for patients with moderate-to-severe active GO, particularly when corticosteroids alone are insufficient. Although teprotumumab, a targeted biologic, has transformed the treatment paradigm [5], this agent is still not available in most countries. Therefore, orbital RT continues to be an essential component in the treatment options for GO. It remains especially valuable for patients who are not candidates for biologic therapies or fail to respond to medical treatment [6]. The primary mechanism of RT involves its non-specific anti-inflammatory effects, which suppress radiosensitive lymphocytes, as well as inhibit fibroblast proliferation and glycosaminoglycan secretion [7].

Despite its positive therapeutic effects, complications have also been reported after orbital RT. These complications include acute exacerbation of soft tissue symptoms and the development of conditions such as dry eye syndrome, cataract, radiation retinopathy, and secondary tumours [8, 9]. However, the majority of studies present sporadic results at the level of individual hospitals, lacking comprehensive reports based on extensive data collection. Furthermore, existing reports predominantly focus on short-term complications, with limited documentation on potential long-term adverse effects. Therefore, there is a need for an analysis using long-term observational data derived from large-scale datasets.

The purpose of this study was to investigate the occurrence of post-RT complications in Korean patients with GO through a nationwide, large-scale cohort study. Additionally, the study aimed to explore factors predictive of radiation retinopathy, critical complication.

## Materials and methods

### Data source

A retrospective cohort study was conducted using customised cohort data obtained from the National Health Insurance Service (NHIS) database (Research Management No. NHIS-2023-1-166). The NHIS database is created from medical expense claims submitted by healthcare providers on behalf of participants. South Korea’s universal healthcare system provides coverage to approximately 98% of its population; consequently, the NHIS has medical information for almost the entire Korean population. The NHIS also regularly provides free, standardised health checkups for all insured people. We obtained customised data on patients with GO from the NHIS database. The customised cohort included patients diagnosed with GO between 2008 and 2018, totalling 44,833 individuals who were followed up until 2020.

This study received an exemption from review by the Institutional Review Board of Chung-Ang University Hospital (IRB No.2401-006-19504), and the requirement for informed consent was waived considering its retrospective design. The study adhered to the guidelines of the Declaration of Helsinki and to the Strengthening the Reporting of Observational Studies in Epidemiology (STROBE) reporting guidelines.

### Study population

**Fig. 1:**
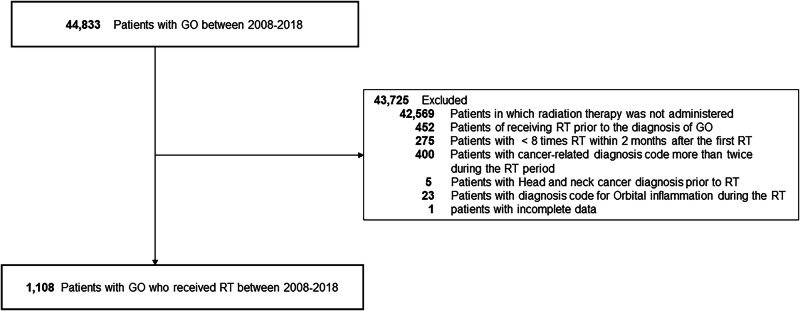
Flow chart of case inclusion.

The diagnosis of GO was confirmed based on the International Classification of Diseases (ICD)-10 code. RT was identified using RT-related claim codes. Each billing code was counted as one fraction, and RT exposure was defined as receiving at least eight fractions within a 2-month period following the initial RT. Exclusion criteria included patients who had received RT before their GO diagnosis, patients who had undergone RT fewer than eight times within 2 months following the initial RT, cases where cancer-related diagnosis codes were duplicated more than twice during the RT period, patients with a prior diagnosis of head and neck cancer before the start of RT, and individuals with a duplicated diagnosis code for orbital inflammation during RT (Fig. 1).

### Definition of outcomes and variables

Post-RT complications were defined as the occurrence of a specific disease that was not present prior to the administration of RT. These complications were identified through the utilisation of ICD-10 diagnostic codes. Complications such as dry eye syndrome, ocular surface disease, and eyelid inflammation were confirmed based on instances in which a diagnostic code was validated on more than two occasions within a 6-month period preceding the administration of RT.

For the assessment of baseline characteristics, we used health information provided by the NHIS. This dataset comprised survey responses and screening measurements collected prior to the initiation of RT. Based on the income decile, individuals in the top ~30%, those in the bottom ~30%, and the remainder were categorised as having high-income, low-income, and middle-income, respectively. Residence was classified into three categories: capital, metropolitan, and rural. Smoking status was divided into nonsmoker and ex- or current smoker. Alcohol consumption was categorised as none or drinker. Body mass index (BMI) was calculated by dividing weight (kg) by height squared (m^2^). Blood glucose (mg/dL) and total cholesterol (mg/dL) were measured using blood drawn after an overnight fast. Baseline systolic blood pressure (SBP) and diastolic blood pressure (DBP) were measured in mmHg. Thyroid disease status was classified into three categories: none, hyperthyroidism, and hypothyroidism. Comorbidities such as diabetes mellitus (DM), hyperlipidaemia, hypertension, autoimmune disease, cardiovascular disease (CVD), and cerebrovascular disease (CBD) were identified using their diagnostic codes.

Intravenous (IV) steroid treatment was defined as the receipt of IV steroids more than six times within 3 months from the diagnosis of GO until the completion of RT. Antithyroid drugs were categorised as none, carbimazole, propylthiouracil, and methimazole. The codes used to identify diseases and interventions are presented in the Supplementary Table (Table S1).

### Statistical analysis

Baseline demographic and clinical characteristics are presented as the median (interquartile range (IQR)) for continuous variables and the percentage for categorical variables. The normality of continuous variables was evaluated using the Shapiro-Wilk test. The Mann-Whitney U test was used to compare continuous variables, and the chi-square test or Fisher’s exact test was employed to compare categorical variables. Since there were missing data in the health examination data, multiple imputation with chained equations was employed to generate 10 imputed datasets. Predictive mean matching, logistic regression, and polytomous regression were used for continuous data, binary data, and categorical data, respectively. A total of 15 univariable and multivariable logistic regression models were used to estimate odds ratios for the effects of risk factors on the occurrence of retinopathy after RT, including baseline demographic and behavioural variables and comorbidities. The odds ratio estimate and its standard error were combined using Rubin’s rule. A total of 16 analyses were conducted using SAS software version 7.0 (SAS Institute, Cary, NC, USA) and R version 4.0.1 (R Foundation for Statistical Computing). For all analyses, a p-value of less than 0.05 was considered statistically significant.

## Results

Between 2008 and 2018, a total of 1108 patients with GO received RT, comprising 458 males and 650 females. The median age of these patients at the time of RT was 51.0 years (IQR, 43.0–59.0), with 729 patients (65.8%) aged between 35 and 59 years. The median follow-up duration following RT was 1215 days (IQR, 781–1859).

Post-RT, 67 patients (6.0%) developed new-onset cataracts, of whom 42 patients (3.8%) subsequently underwent cataract surgery. Furthermore, 226 patients had cataracts prior to receiving RT, of whom 37 patients (16.4%) subsequently underwent cataract surgery following treatment. Radiation retinopathy was identified in 63 patients (5.7%). Prior to RT, dry eye syndrome was observed in 859 out of 1108 patients, indicating a prevalence of 77.5%. Following RT, 37 patients (3.3%) had newly developed dry eye syndrome. Additionally, other complications were noted, including ocular surface disease in 14 patients (1.3%), eyelid inflammation in 8 patients (0.7%), and head and neck cancer in 2 patients (0.2%). Notably, no cases of leukaemia or lymphoma were reported (Table 1). Among the types of ocular surface diseases, keratoconjunctivitis was the most frequently observed, with 9 cases documented.

**Table 1:** Incidence of complications after orbital radiotherapy.

Outcome	Number of patients (%)
Cataract	67 (6.0%)
Cataract surgery	
Newly diagnosed and underwent surgery	42 (3.8%)
Previously diagnosed and underwent surgery	37 (3.3%)
Radiation retinopathy	63 (5.7%)
Dry eye syndrome	37 (3.3%)
Ocular surface disease	14 (1.3%)
Eyelid inflammation	8 (0.7%)
Head and neck cancer	2 (0.2%)
Leukaemia/lymphoma	0 (0%)

Radiation retinopathy was diagnosed at a median of 605 days (IQR, 168–971) following RT, with the latest onset occurring at 3221 days post-treatment. Table 2 shows a comparison of the characteristics of patients with and without radiation retinopathy. In comparison with patients without radiation retinopathy, patients diagnosed with radiation retinopathy were older at the time of GO diagnosis (*p* = 0.044), had a shorter interval from GO diagnosis to RT (*p* < 0.001), exhibited higher fasting blood glucose (FBS) levels (*p* = 0.013), and had a greater prevalence of CVD or CBD (*p* = 0.05). The frequency of radiation retinopathy was higher in patients with pre-existing diabetic retinopathy (9.5%) who received orbital radiation than in those without (5.6%), but the difference was not statistically significant (*p* = 0.171).

**Table 2:** Comparison of baseline characteristics between GO patients with and without radiation retinopathy after RT.

Characteristic	No radiation retinopathy	Radiation retinopathy	Total	*p*-value	Missing (%)
	(*N* = 1045)	(*N* = 63)	(*N* = 1108)		
Age at RT (years), n (%)				0.909	
< 35	72 (6.9%)	3 (4.8%)	75 (6.8%)		
35–59	687 (65.7%)	42 (66.7%)	729 (65.8%)		
$\,\ge$ 60	286 (27.4%)	18 (28.6%)	304 (27.4%)		
Age with GO (years), median [IQR]	51.0 [43.0–59.0]	55.0 [45.5–61.0]	51.0 [43.0–59.0]	**0.044**	
GO–RT period (months), median [IQR]	6.5 [2.7–16.7]	3.7 [1.8–7.9]	6.2 [2.6–15.6]	**< 0.001**	
Sex, n (%)				0.904	
Male	431 (41.2%)	27 (42.9%)	458 (41.3%)		
Female	614 (58.8%)	36 (57.1%)	650 (58.7%)		
Income, n (%)				0.783	20 (1.8%)
Low	212 (20.7%)	15 (24.2%)	227 (20.9%)		
Middle	325 (31.7%)	18 (29.0%)	343 (31.5%)		
High	489 (47.7%)	29 (46.8%)	518 (47.6%)		
Region, n (%)				0.396	
Capital	578 (55.3%)	40 (63.5%)	618 (55.8%)		
Metropolitan	177 (16.9%)	10 (15.9%)	187 (16.9%)		
Rural	290 (27.8%)	13 (20.6%)	303 (27.3%)		
Smoking status, n (%)				0.722	187 (16.9%)
None	521 (60.0%)	33 (63.5%)	554 (60.2%)		
Current or ex-smoker	348 (40.0%)	19 (36.5%)	367 (39.8%)		
Drinking, n (%)				0.915	187 (16.9%)
None	550 (63.3%)	32 (61.5%)	582 (63.2%)		
Drinker	319 (36.7%)	20 (38.5%)	339 (36.8%)		
BMI, median [IQR]	23.1 [21.1–25.2]	23.1 [22.0–25.6]	23.1 [21.1–25.3]	0.281	186 (16.8%)
FBS (mg/dL), median [IQR]	96.0 [88.0–105.0]	100.0 [91.5–107.0]	96.0 [88.0–105.0]	**0.013**	186 (16.8%)
SBP, median [IQR]	120.0 [110.0–130.0]	122.0 [113.5–130.0]	120.0 [110.0–130.0]	0.696	186 (16.8%)
DBP, median [IQR]	75.0 [70.0–80.0]	75.5 [69.0–80.0]	75.0 [70.0–80.0]	0.948	186 (16.8%)
Total cholesterol (mg/dL), median [IQR]	182.0 [158.0–211.0]	183.0 [151.5–210.5]	182.5 [158.0–211.0]	0.49	192 (17.3%)
DM, n (%)	210 (20.1%)	15 (23.8%)	225 (20.3%)	0.579	
Hyperlipidaemia, n (%)	400 (38.2%)	24 (38.1%)	424 (38.2%)	1	
Hypertension, n (%)	365 (34.9%)	23 (36.5%)	388 (35.0%)	0.901	
Autoimmune disease, n (%)	183 (17.5%)	11 (17.5%)	194 (17.5%)	1	
CVD or CBD, n (%)	41 (3.9%)	6 (9.5%)	47 (4.2%)	**0.045**	
Diabetic retinopathy	58 (5.6%)	6 (9.5%)	64 (5.8%)	0.171	
IV steroids, n (%)	519 (49.6%)	29 (46.0%)	548 (49.4%)	0.672	
Pseudophakia, n (%)	58 (5.5%)	7 (11.1%)	65 (5.9%)	0.089	
Antithyroid drugs, n (%)				0.716	
None	168 (16.1%)	12 (19.0%)	180 (16.2%)		
Carbimazole	74 (7.1%)	6 (9.5%)	80 (7.2%)		
Propylthiouracil	124 (11.9%)	6 (9.5%)	130 (11.7%)		
Methimazole	679 (65.0%)	39 (61.9%)	718 (64.8%)		
Thyroid disease status, n (%)				0.055	
None	40 (3.8%)	5 (7.9%)	45 (4.1%)		
Hyperthyroidism	972 (93.0%)	54 (85.7%)	1026 (92.6%)		
Hypothyroidism	33 (3.2%)	4 (6.3%)	37 (3.3%)		

*GO* Graves’ orbitopathy, *RT* radiotherapy, *BMI* body mass index, *FBS* fasting blood sugar, *SBP* systolic blood pressure, *DBP* diastolic blood pressure, *DM* diabetes mellites, *CVD* cardiovascular disease, *CBD* cerebrovascular disease, *IV* intravenous, *n* number, *IQR* interquartile range.

Univariable logistic regression analysis indicated that a shorter duration from GO diagnosis to RT (OR = 0.95, 95% CI = 0.93–0.98, *p* = 0.003), elevated FBS levels (OR = 1.01, 95% CI = 1.00–1.02, *p* = 0.017), and the presence of CVD or CBD (OR = 2.58, 95% CI = 1.05–6.33, *p* = 0.038) were significantly associated with the occurrence of radiation retinopathy. In a multivariable logistic regression analysis, both a shorter duration from GO diagnosis to RT (OR = 0.95, 95% CI = 0.92–0.98, *p* = 0.003) and higher FBS levels (OR = 1.01, 95% CI = 1.00–1.03, *p* = 0.014) remained significantly associated with radiation retinopathy (Table 3).

**Table 3:** Logistic regression analyses for the risk factors associated with radiation retinopathy.

Variable	Univariable		Multivariable	
	Crude OR (95% CI)	*p*-value	Adjusted OR (95% CI)	*p*-value
Age at RT (years)				
<35	1 (Reference)		1 (Reference)	
35–59	1.47 (0.44,4.85)	0.53	1.29 (0.37,4.54)	0.687
$\,\ge$60	1.51 (0.43,5.27)	0.517	0.87 (0.21,3.62)	0.845
GO–RT period (months)	0.95 (0.93,0.98)	**0.003**	0.95 (0.92,0.98)	**0.003**
Sex				
Male	1 (Reference)		1 (Reference)	
Female	0.94 (0.56,1.56)	0.801	0.81 (0.36,1.82)	0.59
Income				
Low	1 (Reference)		1 (Reference)	
Middle	0.8 (0.39,1.62)	0.524	0.66 (0.31,1.42)	0.273
High	0.83 (0.44,1.58)	0.567	0.70 (0.36,1.37)	0.292
Region				
Capital	1 (Reference)		1 (Reference)	
Metropolitan	0.82 (0.4,1.67)	0.577	0.8 (0.38,1.69)	0.559
Rural	0.65 (0.34,1.23)	0.184	0.58 (0.3,1.13)	0.109
Smoking status				
None	1 (Reference)		1 (Reference)	
Current or Ex-smoker	0.79 (0.45,1.37)	0.376	0.54 (0.23,1.26)	0.128
Drinking				
None	1 (Reference)		1 (Reference)	
Drinker	1.05 (0.58,1.89)	0.855	1.21 (0.61,2.38)	0.537
BMI	1.04 (0.96,1.14)	0.279	1.05 (0.95,1.17)	0.249
FBS	1.01 (1.00,1.02)	**0.019**	1.01 (1.00,1.03)	**0.011**
SBP	1 (0.98,1.02)	0.811	0.99 (0.96,1.02)	0.561
DBP	0.99 (0.97,1.02)	0.679	1 (0.95,1.04)	0.867
Total cholesterol	1 (0.99,1.01)	0.481	1 (0.99,1)	0.402
DM	1.24 (0.68,2.26)	0.477	0.85 (0.36,1.96)	0.693
Hyperlipidaemia	0.99 (0.59,1.68)	0.977	0.99 (0.55,1.78)	0.966
Hypertension	1.07 (0.63,1.82)	0.799	0.92 (0.48,1.77)	0.797
Autoimmune disease	1 (0.51,1.95)	0.992	0.93 (0.43,1.99)	0.848
CVD or CBD	2.58 (1.05,6.32)	**0.039**	2.28 (0.81,6.4)	0.118
IV steroids	0.86 (0.52,1.44)	0.576	0.87 (0.5,1.51)	0.629
Pseudophakia	2.13 (0.93,4.87)	0.074	2.27 (0.88,5.88)	0.088
Diabetic retinopathy	1.79 (0.74,4.33)	0.195	1.08 (0.34,3.26)	0.934
Antithyroid drugs				
None	1 (Reference)		1 (Reference)	
Carbimazole	1.14 (0.41,3.14)	0.807	2.29 (0.61,8.57)	0.217
Propylthiouracil	0.68 (0.25,1.85)	0.448	1.22 (0.33,4.56)	0.768
Methimazole	0.8 (0.41,1.57)	0.523	1.44 (0.51,4.08)	0.493
Thyroid disease status				
None	1 (Reference)		1 (Reference)	
Hyperthyroidism	0.44 (0.17,1.17)	0.101	0.33 (0.09,1.22)	0.096
Hypothyroidism	0.97 (0.24,3.91)	0.965	0.8 (0.17,3.71)	0.775

*OR* odds ratio, *GO* Graves’ orbitopathy, *RT* radiotherapy, *BMI* body mass index, *FBS* fasting blood sugar, *SBP* systolic blood pressure, *DBP* diastolic blood pressure, *DM* diabetes mellitus, *CVD* cardiovascular disease, *CBD* cerebrovascular disease, *IV* intravenous.

## Discussion

In this study, we examined the data of 1108 patients diagnosed with GO who received RT between 2008 and 2018, monitoring their progress for a minimum duration of 2 years to determine the incidence of complications that may arise following RT. The results revealed that 6% of the patients had newly developed cataracts, 5.7% of them had radiation retinopathy, and 3.3% of them suffered from newly developed dry eye syndrome. Notably, only 2 cases (0.2%) of head and neck cancer were reported. We identified significant risk factors for radiation retinopathy, which included a short interval from GO diagnosis to RT and elevated FBS levels. Although the potential complications associated with RT have been documented in several studies, the reported incidence rates have shown considerable variability. This study is distinctive in that it not only confirms the incidence of these complications in a substantial cohort but also elucidates the risk factors associated with the development of radiation retinopathy by analysing the comprehensive health profiles of the participants.

Radiation retinopathy is characterised as a progressive microangiopathy that results in the localised loss of capillary endothelial cells and adjacent cellular structures. The reported incidence of radiation retinopathy following RT is generally considered to be quite low, with various studies indicating an incidence rate of 1% or less [10–14]. However, our study identified 63 patients (5.7%) with radiation retinopathy, which appears to be higher than previously anticipated. This discrepancy may be attributed to the assessment of patients for this condition only if they had visual impairment in previous studies. Such selective evaluation may explain the relatively lower incidence of radiation retinopathy reported in retrospective studies compared to prospective ones. In a prospective study, it was observed that 4 out of 42 patients (9.52%) exhibited retinal microvascular abnormalities [15]. Additionally, another study indicated that 21% of patients met the criteria for possible retinopathy, characterised by the presence of one or more retinal haemorrhages or microaneurysms [16]. Consequently, it appears that the incidence of microvascular abnormalities following RT may be higher than previously anticipated; however, the reported incidence of radiation retinopathy may fluctuate based on the specific diagnostic criteria employed. Our study relied solely on diagnostic codes submitted to the NHIS, which limit our ability to determine whether diagnostic criteria were applied uniformly across the cases. Furthermore, recent advancements in various diagnostic techniques may have contributed to the observed increase in the incidence of radiation retinopathy in our study. Given that our NHIS customised dataset was generated based on predefined variables, procedure-related information, such as laser photocoagulation or intravitreal anti-vascular endothelial growth factor treatment, was not available for severity stratification.

The findings of our study indicated that a shorter duration of disease from GO diagnosis to the initiation of RT and elevated FBS levels were significantly correlated with the onset of radiation retinopathy following RT. Despite achieving statistical significance, the adjusted effect sizes were small (GO–RT interval: OR = 0.95, 95% CI = 0.92–0.98; FBS: OR = 1.01, 95% CI = 1.00–1.03), indicating modest effects at the individual level; thus, these findings should be interpreted with caution. Notably, the association between a shorter disease duration and the development of radiation retinopathy suggests that RT may exacerbate vascular fragility during the early and active stages of GO, which is characterised by severe active inflammation, thereby increasing the risk of retinal complications in affected patients. Recent studies have reported the effectiveness of a combination of systemic steroids and RT in managing active GO [17, 18].

Previous studies have established that individuals with DM and diabetic retinopathy have an elevated risk of developing radiation retinopathy following RT [10, 19]. However, in the current study, DM alone did not emerge as a significant risk factor for radiation retinopathy. Instead, FBS elevation was significantly associated with the development of radiation retinopathy. Therefore, compared to well-managed DM, uncontrolled hyperglycaemia may exert a more substantial impact on retinal vascular cells, leading to more pronounced alterations in retinal capillaries than would typically be anticipated from the mere presence of diabetic disease.

Hypertension has been recognised as a potential risk factor for radiation retinopathy [20]. However, in the present study, hypertension did not demonstrate a statistically significant association with radiation retinopathy. Conversely, CVD and CBD were identified as significant risk factors for retinopathy in the univariable logistic regression model. Nevertheless, this association was weakened in the multivariable model and did not achieve statistical significance (*p* = 0.082). This finding suggests the trending significance of CVD and CBD. Patients with pre-existing vascular conditions may have an increased risk of retinal vessel compromise, which may exacerbate the ischemic changes in the retina induced by radiation.

The timeline for the onset of radiation retinopathy following RT remains uncertain. Cases of radiation retinopathy have been documented occurring more than 15 years after the administration of a standard dose of 20 Gy delivered in fractionated doses [21]. In the present study, the median duration from RT to the onset of radiation retinopathy was 1.7 years, with some cases of onset occurring as late as 9 years after treatment. Therefore, it is vital to conduct follow-up and monitoring for a sufficient duration following RT.

Following RT, 67 patients (6.0%) had newly developed cataracts, with 42 patients (3.8%) subsequently undergoing cataract surgery. Additionally, among the 226 patients with pre-existing cataracts prior to RT, 37 individuals required surgical intervention. These findings indicate that RT not only contributes to the onset of cataracts but may also aggravate preexisting conditions. Prior studies have documented cataract incidence rates of 10% to 11% in patients with GO following RT, with the average time from radiation exposure to cataract development estimated at approximately 2 years; however, this interval can range from a minimum of 6 months to over 10 years [10, 22]. The occurrence of cataracts is influenced by various factors, including age and the use of steroids, necessitating a cautious interpretation of the results. It is important to note that cataracts may not initially present a significant threat to vision; thus, vigilant post-RT monitoring and follow-up over an appropriate duration are essential.

In our study, the incidence rates of dry eye and ocular surface disease were 3.3% and 1.3%, respectively, following orbital RT for GO. These percentages are significantly lower than those reported in earlier studies, which presented dry eye incidence rates ranging from 47% to 81% [23, 24]. A significant proportion of patients with GO already have dry eye or ocular surface disease, and exposure to radiation may induce inflammatory changes in the conjunctiva and lacrimal glands, potentially further compromising the integrity of the ocular surface. The discrepancy between our findings and those of prior studies may be attributed to our focus on newly diagnosed cases without a previous history of dry eye or ocular surface disease. In this study, it was observed that 77.5% of patients who underwent RT had been diagnosed with dry eye syndrome within 6 months preceding the initiation of RT. Furthermore, variations in follow-up durations and the diagnostic criteria used for dry eye and ocular surface disease across different studies may explain the differences in incidence rates. Our findings underscore the necessity for tailored management approaches and rigorous monitoring of the ocular surface in GO patients undergoing RT.

In the present study, 2 patients (0.2%) were diagnosed with head and neck cancer, and no cases of leukaemia or lymphoma were reported. Both tumours arose in the oral and pharyngeal regions, which lie outside the orbital radiation field, making a causal link to orbital radiotherapy unlikely. Some studies have reported an overall low incidence of cancer following RT [25–27]. However, other studies have estimated a theoretical lifetime risk of radiation-induced malignancies of approximately 0.3% to 1.2% [9, 28]. The present study did not calculate lifetime cumulative risk for radiation retinopathy or secondary cancer. Although RT has been suggested to be contraindicated for individuals under the age of 35 years due to the potential risk of long-term tumour development [29], evidence remains limited and inconclusive. Therefore, decisions for younger patients should be individualised, with counselling on potential long-term risks and available alternatives. In the study cohort of 1108 patients who received RT, 75 patients were younger than 35 years. Importantly, the incidence of radiation retinopathy in this age group (4.0%) was comparable to that observed among older patients, indicating that younger age did not confer an increased risk of this complication in our dataset. Additional data regarding the appropriate age threshold for administering RT are required, necessitating long-term follow-up studies in the future.

The strengths of this study lie in its extended follow-up duration and large cohort size, which facilitate the robust detection of complications. However, the retrospective design of the study limits our ability to establish causal relationships as the data can only identify risk factors associated with the development of radiation retinopathy. Furthermore, the study relied on insurance data that comprised diagnosis codes, which may be subject to inaccuracies. As the database does not provide information on the delivered dose and clinical fraction size, the accumulated radiation dose could not be determined. Additionally, our cohort may not fully represent all ethnic or demographic groups, limiting the generalisability of the findings.

In conclusion, this study utilised big data from the NHIS to assess the incidence of various complications following RT in patients with GO. The incidence of radiation retinopathy was found to be 5.7%. Patients with a short intervals between the diagnosis of GO and initiation of RT and those with elevated FBS levels should be carefully screened and monitored for the development of radiation retinopathy. It is crucial to understand the true risks and benefits of RT so that this valuable treatment modality may be used appropriately and safely.

## Summary

### What was known before

- In cases of Graves’ orbitopathy (GO), orbital radiotherapy (RT) is a useful second-line treatment for patients unresponsive to corticosteroids or unsuitable for biologic therapies.
- While the incidence of radiation retinopathy following RT is generally low, it exhibits considerable variability influenced by the diagnostic criteria employed, the timing of diagnosis, and the methodologies utilised in each study.

### What this study adds

- After orbital RT, new adverse events that emerged included cataract (6.0%), and, less frequently, dry eye-related or inflammatory conditions and malignancy.
- Among patients with GO, the incidence of radiation retinopathy after RT was 5.7%, with risk factors including a short interval between the diagnosis of GO and RT and elevated fasting blood glucose levels.

## Supplementary information

Table S1. Codes used for defining the study population, intervention, outcomes, and comorbidities.

## Data Availability

The data that support the findings of this study are available from the National Health Insurance Service (NHIS) of Korea, but restrictions apply to their availability. These data were used under license for the current study and are not publicly available. Data are, however, available form the authors upon reasonable request and with permission of the NHIS.
