# Supplementary material for: Complications after radiotherapy in patients with Graves’ orbitopathy: A nationwide cohort study
Source: Eye (Lond). 2026 Feb 21;40(7):1067–73. doi: 10.1038/s41433-026-04284-9 (PMC13161218; doi:10.1038/s41433-026-04284-9)
Supplement: Supplementary file 1 — Table S1. Codes used for defining the study population, intervention, outcomes, and comorbidities. [file 41433_2026_4284_MOESM1_ESM.docx]

Table S1. Codes used for defining the study population, intervention, outcomes, and comorbidities

|  | Code |
| --- | --- |
| Graves’ orbitopathy | H062 |
| Radiotherapy | HD051, HD052, HD053, HD054, HD055, HD056, HD057, HD058, HD059, HD061, HD110, HZ271 |
| Cataract | H25, H262, H268, H269 |
| Cataract surgery | S5119 |
| Radiation retinopathy | H358, H359 |
| Dry eye syndrome | H0411 |
| Ocular surface disease | H160 (corneal ulcer), H1611 (filamentary keratitis), H1613 (punctate keratitis), H1618 (superficial keratitis), H162 (keratoconjunctivitis), H168 (other keratitis), H169 (unspecified keratitis), H17 (corneal scar and opacity), H182 (corneal edema), H184 (corneal degeneration) |
| Eyelid inflammation | H01 |
| Head and neck cancer | C00-C14, C760, C770 |
| Leukemia/lymphoma | C81-C96 |
| Thyroid cancer | C73 |
| Hypothyroidism | E02, E03 |
| Hyperthyroidism | E05 |
| Autoimmune disease | D271 (autoimmune adrenalitis), D510 (pernicious anemia), D591 (autoimmune hemolytic anemia), D86 (sarcoidosis), E10 (type Ⅰ diabetes mellitus), G35 (multiple sclerosis), G700 (myasthenia gravis), L80 (vitiligo), M06 (rheumatoid arthritis), M32 (systemic lupus erythematous), M350 (Sjogren syndrome), M353 (polymyalgia rheumatica) |
| Diabetes mellitus | E10-E14 |
| Hyperlipidemia | E78 |
| Hypertension | I10-I15 |
| Cardiovascular disease | I20-I25 |
| Cerebrovascular disease | I60-I69 |
| Pseudophakia | Z961, S5119 |
| Diabetic retinopathy | H360 |
| Orbital inflammation | H050, H051 |
| IV steroid | 193601BIJ, 193602BIJ, 193603BIJ, 193604BIJ |
| Carbimazole | 471501ATB, 471502ATB, 471503ATB |
| Propylthiouracil | 220101ATB, 220102ATB |
| Methimazole | 191801ATB, 191802ATB, 191803ATB |

IV, intravenous
